# Bioactivity and Component Analysis of Water Extract of *Sophora japonica* against Hyperuricemia by Inhibiting Xanthine Oxidase Activity

**DOI:** 10.3390/foods11233772

**Published:** 2022-11-23

**Authors:** Shunyi Jiang, Danni Song, Honghui Zhao, Fuqi Wang, Xin Su, Xinyang Zhang, Xu Zhao

**Affiliations:** 1 Faculty of Functional Food and Wine, Shenyang Pharmaceutical University, Shenyang 110016, China; 2 School of Traditional Chinese Material Medica, Shenyang Pharmaceutical University, Shenyang 110016, China

**Keywords:** *sophora japonica*, xanthine oxidase, bioactivity, component analysis, flavonoids and polyphenols

## Abstract

Hyperuricemia (HUA) is a metabolic condition caused by excessive production or low excretion of uric acid (UA) in the body. Xanthine oxidase (XOD) is the key enzyme in the process of metabolism purines to generate UA. In this study, the in vitro inhibitory effect of water extract of the flower bud of *Sophora japonica* (WESJ) on XOD was investigated by ultraviolet spectrophotometry. A mice model of HUA was constructed to explore the effect of WESJ on UA levels and the mechanism of action on renal function. Based on Box–Behnken design, the optimal extraction process of WESJ was determined to extract *Sophora japonica* twice with 8 times of water, 0.5 h each time. Pharmacological results showed that low, medium, and high doses of WESJ (200, 400, 600 mg/kg) could significantly reduce serum UA level, inhibit the activity of XOD in blood and liver, and have a protective effect on kidney damage caused by high UA. Through UPLC-Q-TOF-MS/MS analysis, 214 compounds were identified in WESJ, including flavonoids, polyphenols, triterpenoids, organic acids, and others. The rat serum of WESJ was analyzed, and 23 prototype components entering the blood were identified, including 15 flavonoids and polyphenols, which may be the main bioactive components. In conclusion, flavonoids and polyphenols in WESJ may reduce the level of UA and alleviate kidney damage by inhibiting the activity of XOD. WESJ is expected to be used as a plant-based food and dietary supplement for the treatment of HUA.

## 1. Introduction

Hyperuricemia (HUA) is a metabolic condition in which the production process or excretion of serum uric acid (UA) is unbalanced in the human body due to long-term active purine metabolism [1,2]. With the gradual improvement of living standards, the incidence of HUA is also increasing year by year, and it has become the second largest metabolic disease, which seriously threatens human health. The pathogenesis of primary hyperuricemia is related to excessive UA production, which may be related to the increase in the number and activity of enzymes in the promotion of UA production pathway or the decrease in the number and activity of enzymes in the inhibition of UA production pathway. Sustained high levels of UA in the human body may lead to relating complications, such as hypertension, heart disease, chronic renal dysfunction, dysglycemia and hyperlipidemia, and other metabolic-related diseases [3,4,5,6].

XOD inhibitors are effective drugs for the treatment of HUA which could hold back the production of UA and reduce its level. Allopurinol is a classic XOD inhibitor. Its mechanism is mainly to competitively inhibit XOD activity and prevent the transformation of UA from xanthine and hypoxanthine to decrease the total metabolism of UA [7,8,9]. However, allopurinol is linked to adverse reactions such as skin allergies, hepatotoxicity, and bone marrow toxicity. Therefore, developing more effective XOD inhibitors with less side effects has become a strategic target for the treatment of HUA [10,11].

*Sophora japonica* is a common medicinal and edible homologous plant with a long history of plant-based food and diet therapy in China. Water extract of the flower bud of *Sophora japonica* (WESJ) contains a variety of natural compounds, mainly including flavonoids, saponins, volatile oils, phenolic acids, and so on [12,13,14]. Polyphenols are the most widely distributed secondary metabolites from plants in dietary sources. Its main activities include anti-oxidation, anti-inflammatory, anti-cancer, and good therapeutic effect on metabolic syndrome [15,16,17,18]. Flavonoid compounds are found in plant foods during our daily life. Flavonoids are derived compounds with variable phenolic structures, so they have similar pharmacological activities to polyphenols [19,20,21]. Based on the literature and our previous research, WESJ shows numerous pharmacological activities, and may have the potential to reduce UA [22,23,24].

In this study, an ultraviolet (UV) spectrophotometry method was developed and optimized to determine the inhibition rate of WESJ on XOD. The optimal extraction process of WESJ was studied with inhibition rate as the dependent variable based on Box–Behnken design (BBD). BBD investigates the interaction of multiple factors and levels to make the results obtained under the optimal conditions more objective. Therefore, BBD is often used to optimize the extraction process and determine the parameters [25,26]. Then, the improvement effect of WESJ on UA level and renal function in HUA model mice and its possible mechanisms were investigated. The chemical components in WESJ were identified by UPLC-Q-TOF-MS/MS, and the serum pharmacochemistry method was utilized to identify the WESJ blood prototype components. We hope that this plant-based food and its principal active components have the potential to be used as drugs or dietary supplements for HUA treatment, and the established UV spectrophotometry for the determination of XOD inhibition could also be relevant for the activity screening of other traditional Chinese medicine and plant-based foods.

## 2. Materials and Methods

### 2.1. Materials

*Sophora japonica* flower buds were supplied by Nanjing Tongrentang Pharmacy (Shenyang, China). Allopurinol, XOD and Tris-HCl were provided by Dalian Meilun Biotechnology Co., Ltd. (Dalian, China). Nitroblue tetrazolium (NBT) was purchased from Youwei Biotechnology Co., Ltd. (Shenyang, China). Hypoxanthine and potassium oxonate were supplied by Shanghai Shenghong Biological Technology Co., Ltd. (Shanghai, China). Other assay kits were provided by Nanjing Jiancheng Bioengineering Institute (Nanjing, China).

### 2.2. Extraction Process of Sophora japonica

*Sophora japonica* flower bud was weighed and soaked with different volumes of water (6×, 8×, 10×) for ultrasonic extraction (80 kHz, KQ-250E, Kun Shan Ultrasonic Instruments Co., Ltd., Kunshan, China). The extraction time was investigated for 0.5, 1.0, and 2.0 h, respectively. Extraction times were explored, that is, the extraction operation was repeated after standing for 0.5 h. The extract was filtered and collected, and the residue was washed with a small amount of water. The detergent was associated with the extract, and all was transferred after concentration, freeze-dried (Scientz-10YD/B, Scientz Biotechnology Co., Ltd, Ningbo, China), sealed, and dried for preservation.

### 2.3. Preparation of WESJ

For UV determination, 3.0 g of *Sophora japonica* flower bud was taken and concentrated to about 1–2 mL in the same extraction process. After freeze-drying, sealing, drying and storage, the dimethyl sulfoxide (DMSO) solution containing 1.0 g/mL of the crude drug was prepared.

For animal administration: An appropriate amount of WESJ powder was added and dissolved with 0.5% carboxymethylcellulose sodium (CMC-Na) solution to prepare samples with concentrations of 200, 400, and 600 mg/kg.

### 2.4. UV Spectrophotometry and XOD Inhibition Activity of WESJ 

UV spectrophotometry was developed to determine the XOD activity in vitro, with xanthine as a substrate. Briefly, 2 µL of DMSO, 158 µL of Tris-HCl (0.1 mol/L, pH 7.4) buffer solution, 10 µL of XOD, and 20 µL of NBT (final concentration 0.83 mmol/L) was added in order into the 96-well plates and pre-incubated at 37 °C with shaking for 3 min, then 50 µL of xanthine was added into the well and shaken for 3 s to generate uric acid. The absorbance was measured immediately after adding xanthine at a wavelength of 560 nm with a Spectra MAX microplate reader (MAX 190, Molecular Devices, Sunnyvale, CA, USA). The measurement was carried out under a kinetic mode, with recording intervals of 10 s and a reading time of 6 min. The final concentrations of XOD, xanthine, and allopurinol in the reaction system were optimized. The final concentrations of XOD (0.1 mol/L Tris-HCl, pH 7.4) tested were 0.0281, 0.0560, 0.1125, 0.2250, 0.4500 U/mL, and the final concentrations of xanthine (resolved in 125 µmol/L NaOH) tested were 0.1, 0.2, 0.4, 0.8, 1.25 mmol/L. Allopurinol (2 µL in DMSO) was used as a positive drug, and IC_50_ was calculated at the final concentrations of 0.26, 0.52, 1.04, 2.08, 4.16, and 8.32 mmol/L.

The inhibitory effects against XOD were evaluated by the developed UV spectrophotometry. In the reaction system, the final concentration of XOD was 0.2250 U/mL, xanthine was 0.4 mmol/L, allopurinol was 0.5492 mmol/L, and WESJ was 1.0 g/mL (crude drug), respectively. The reaction lasted for 5 min. The enzyme activities of each group were measured by microplate dynamic method (560 nm), and V_max_ was recorded. 

Sample inhibition rate (%) = (A − B)/A × 100% (A as V_max_ in the control group, and B as V_max_ in the sample group).

### 2.5. Animals and Experimental Design

Male Kunming mice (20–25 g) were provided by Liaoning Changsheng Technology Co., Ltd. All animals were allowed to eat standard food and water ad libitum, and adapted to the environment at 22 ± 2 °C and 60 ± 5% humidity with a fixed 12 h light/dark cycle. After acclimating for one week, the male mice were divided into 6 experimental groups randomly with 10 mice in each group: control group (Con), model group (HUA), allopurinol group (ALL), and WESJ groups (Low, Medium, High). Except for Con group, mice in all groups were given oral administration of potassium oxonate (i.g., 500 mg/kg) and hypoxanthine (i.g., 500 mg/kg) to induce HUA for 7 days. Then, Con and HUA groups were treated with 0.5% CMC-Na, and the ALL group was treated with allopurinol (i.g., 5 mg/kg). WESJ groups at three levels were treated with a dose of WESJ at 200, 400, 600 mg/kg by gavage, respectively. 

### 2.6. Biochemical Analysis

One hour after the last dose, the eyeballs were removed to collect the blood, and then the liver and kidney tissues were removed following the execution of the mice. Blood samples were centrifuged at 3000 rpm for 10 min at 4 °C to separate serum. The corresponding biochemical kits were used to determine UA, creatinine (Cr), blood urea nitrogen (BUN) level in serum, and XOD activity in serum and liver. The liver and one kidney tissue were collected and washed with saline, weighed, and temporarily stored at 4 °C for subsequent biochemical analysis. 

### 2.7. Histopathological Examinations

The other side of the kidney was fixed in 4% paraformaldehyde for 24 h. Sections were dehydrated with ethanol, cleared with xylene, and embedded in paraffin with a thickness of 3 µm. After staining with hematoxylin and eosin (H&E), tissue sections were observed using a light microscope.

### 2.8. LC-Q-TOF-MS/MS Detection

#### 2.8.1. LC-Q-TOF-MS/MS Analysis

UPLC separation was carried out on Sciex Exion LC equipped with a binary pump, an auto-sampler, a thermostatically controlled column apartment, and a Phenomenex C18 (2.1 × 100 mm, 2.6 μm). The mobile phases were 0.05% formic acid in water (phase A) and a mixture of methanol and acetonitrile (50:50 *v*/*v*, phase B). The flow rate was 0.3 mL/min and the injection volume was 2.0 μL. The gradient program was used as follows: 0–1.0 min, 5% B; 3.5–18.0 min, 28–98% B; 18.0–22.0 min, 98% B; 22.1–25.0 min, 5% B. 

The eluates from the UPLC system were directly entered into an X500R Q-TOF-MS/MS system. The electrospray ionization (ESI) source was operated with a mass range of 80–1250 *m*/*z* in positive and negative ionization mode, respectively. Ion source temperature was set at 550 °C. Appropriate MS/MS productions were obtained by different collision energies under 20, 40, and 60 eV.

#### 2.8.2. Determination of Chemical and Serum Components of WESJ

The chemical and serum components of WESJ were detected in the above conditions. A small amount of WESJ freeze-dried powder was taken and ultrasonically dissolved by DMSO and methanol–water (50:50, *v*/*v*) before centrifugation. 

Six male SD rats (180–220 g) were provided by Liaoning Changsheng Technology Co., Ltd. Following one week of acclimation, rats received a solution of WESJ by gavage at a dose of 600 mg/kg twice daily. One milliliter of blood was taken 2 h after dosing, and was stored at 4 °C. Serum samples were obtained by centrifuging the blood at 3000 rpm at 4 °C for 10 min. All serum samples were mixed in equal proportions, and the resulting mixture was precipitated two times by adding 90% methanol. After centrifugation, the supernatant was stored after freeze-drying. The residue was resolved with methanol–water (50:50, *v*/*v*) and analyzed with the method described above for the separation and detection of the components of WESJ absorbed into the rat blood circulation.

### 2.9. Data Analysis

Data of pharmacodynamic study were reported as mean ± standard error, using the unpaired Student’s *t*-test. Value of *p* < 0.05 calculated by GraphPad Prism 5.0 was considered significant.

To identify the components of WESJ, the instrument self-configuration TCM MS/MS Library (including 22,938 compounds of more than 3000 Chinese herbal medicines) was searched according to the first-order accurate mass number, isotope distribution ratio and MS/MS of the compounds. In addition, neutral loss identification, ChemSpider database, and MS-DIAL software were used to identify more components of WESJ. With all identified WESJ components as the target, searching for the same components could be absorbed by rats in the serum.

## 3. Results and Discussion

### 3.1. Development of the UV Spectrophotometry

**Figure 1:**
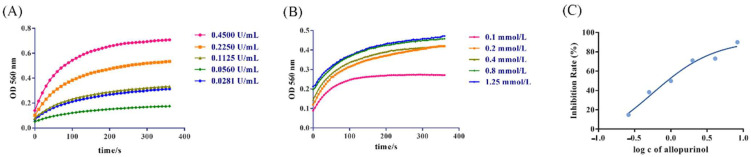
Optimization of XOD catalytic reaction conditions. (**A**) Kinetics of XOD at enzyme concentrations. (**B**) Kinetics of XOD at substrate concentrations. (**C**) Inhibition rate of allopurinol.

In this study, a UV spectrophotometry method was developed to determine the UA produced by the reaction to evaluate the XOD inhibition activity. The principle of the method is that the substrate xanthine or hypoxanthine generates UA and peroxide free radicals under the action of XOD. The free radicals react with the added NBT to produce purple compound methyl, which shows the maximum absorption at 560 nm on a UV spectrophotometer [27,28]. After optimization of the reaction conditions, the result is shown in Figure 1. In the early stages of reactiona, the reaction rate becomes faster with the increase of XOD concentration. Therefore, the optimal concentration (0.2250 U/mL) was selected for the enzyme concentration with a moderate reaction rate, which can improve the accuracy of the reaction and save the cost. When the substrate concentration was above 0.4 mmol/L, the reaction rate would not increase, indicating that the substrate was excessive. Thus, 0.4 mmol/L was selected as the optimal substrate concentration. Allopurinol significantly inhibited the activity of XOD, and when the inhibition rate was 50%, the concentration of allopurinol was the optimal concentration (0.5492 mmol/L). 

### 3.2. Optimization of Extraction Process

**Figure 2:**
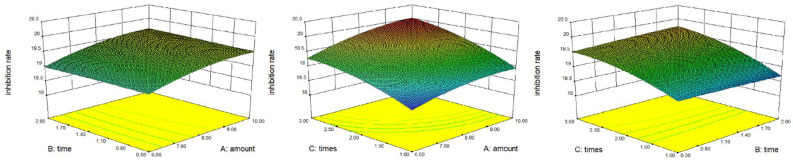
The 3D response surface diagram of dependent variables against independent variables. A: the amount of water; B: extraction time; C: extraction times; (R1) the inhibition rate of XOD.

The model was successfully fitted with the amount of water, extraction time, and extraction times as independent variables, and the XOD inhibition rate of the extract as a dependent variable. Figure 2 shows the 3D response surface diagram. The regression equation is as follows:

R1 = 19.51 + 0.29 × A + 0.037 × B + 0.46 × C + 0.036 × AB + 0.075 × AC + 0.079 × BC − 0.15 × A^2^ − 0.014 × B^2^ − 0.18 × C^2^ (R^2^ = 0.9851)

The experimental data showed that the effect of reaction time on inhibition rate was not significant, and the inhibition rate of each group was similar. Considering the efficiency and cost of the experiment, the optimal extraction process was finally determined to extract *Sophora japonica* twice with 8 times of water, 0.5 h each time. Under the optimum conditions, the extraction rate of WSFJ was 14.8% (*w*/*w*) and the inhibition rate was 19.5%.

### 3.3. Effects of WESJ on Body Weight and Organ Index

**Figure 3:**
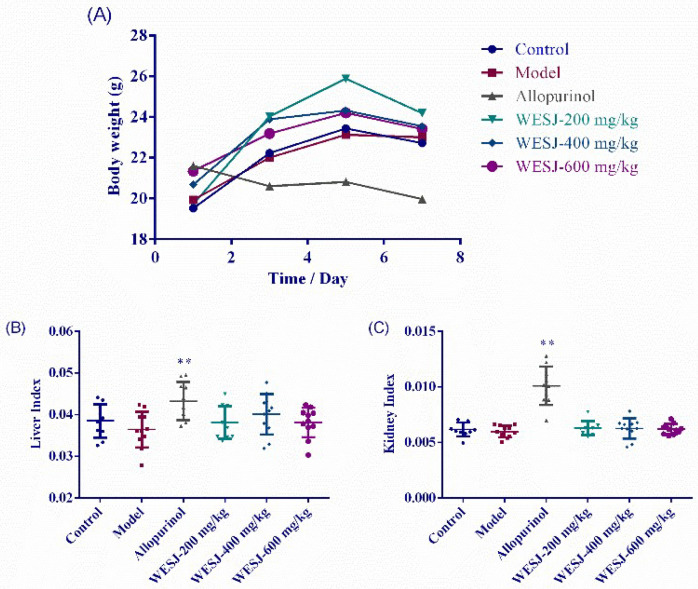
Effects of WESJ on body weight and organ index in hyperuricemic mice. (**A**) Body weight in different groups; (**B**) liver index in different groups; (**C**) kidney index in different groups. Data were expressed as mean ± S.E.M. (*n* = 10). ** *p* < 0.01, compared with model group.

As shown in Figure 3A, except for the allopurinol group, the body weight of other groups showed a common trend of increase. A slight weight loss on the 7th day may be related to fasting the previous day. In the allopurinol group, weight loss was observed daily. Depression and loss of appetite were also observed during feeding, which may be related to the side effects caused by allopurinol [29]. 

Organ index is usually expressed as the ratio of individual organ to body weight. Under normal conditions, the organ index is relatively constant. When an animal is infected, the organ index may change due to the weight of the damaged organ, so this value can be used to assess whether the organ is damaged [30]. As can be seen from Figure 3B,C, compared with other groups, the liver index and kidney index of allopurinol group increased significantly, suggesting that allopurinol may cause hyperplasia and hypertrophy in mouse organs. Abnormal renal appearance was observed during tissue sampling, with swelling and whitening of the kidneys in the allopurinol group. Compared with the model group and allopurinol group, WESJ had no significant effect on the organ index of mice. In general, WESJ did not significantly affect the weight change of hyperuricemic mice, nor did it cause the change of liver and kidney index. 

### 3.4. Biochemical Effects of WESJ on Hyperuricemic Mice

**Figure 4:**
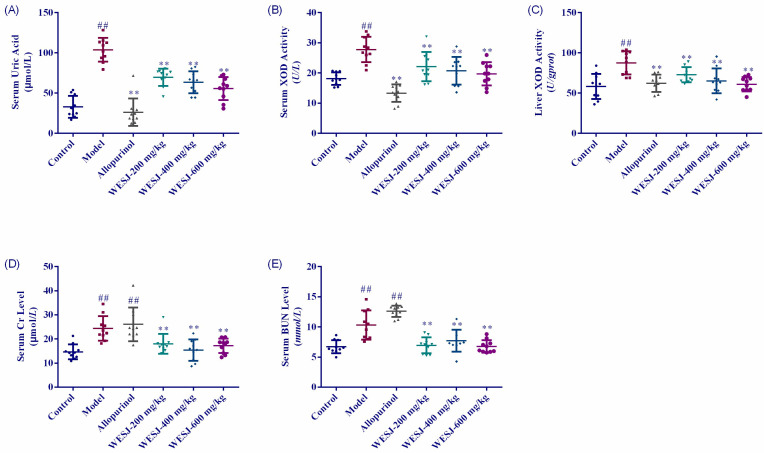
Biochemical effects of WESJ on hyperuricemic mice. (**A**) Levels of UA in serum; (**B**) levels of XOD in serum; (**C**) levels of XOD in the liver; (**D**) levels of Cr in serum; (**E**) levels of BUN in serum. Data were expressed as mean ± S.E.M. (*n* = 10). ## *p* < 0.01, compared with control group; ** *p* < 0.01, compared with model group.

Figure 4 shows the measurement results of biochemical indexes in each group of mice. Serum UA levels in model group were significantly higher than that in control group after 7 consecutive days of modeling treatments (Figure 4A), indicating that the HUA mice model was successfully established. Compared with model group, the UA levels of each WESJ group decreased significantly after 7 days of treatments (*p* < 0.01). Although the effect of reducing uric acid was not as significant as that of allopurinol group, WESJ could reduce UA level in a dose-dependent manner. The effects of WESJ on XOD activity in serum and liver were shown in Figure 4B,C, respectively. The XOD activity in serum and liver of model group was significantly higher than that of the control group. Compared with the model group, XOD activity in low, medium, and high dose WESJ groups decreased markedly, suggesting that WESJ may inhibit the XOD activity in serum and liver so as to reduce the production of UA.

The results of serum Cr and BUN levels are shown in Figure 4D,E. Compared to the control group, serum Cr and BUN levels of mice in model and allopurinol group were markedly increased, suggesting that the renal function of these two groups of mice was impaired. WESJ significantly reversed the levels of these two indicators (*p* < 0.01), indicating that it may improve the renal excretion function of mice.

As indicators of renal function, Cr and BUN can reflect the status of renal function. Cr is a product of muscle metabolism, which is finally filtered through the glomerulus and excreted in the urine. When renal function declines, the levels of Cr increase and Cr clearance decreases. BUN is the final product of protein metabolism. When renal function is abnormal and glomerular filtration rate decreases, BUN levels increase. In addition, the increase of urea nitrogen level is not only the cause of renal insufficiency, but also related to other factors, such as high fever or infection, gastrointestinal bleeding or liver disease, and drug effects [31,32,33,34,35]. In our study, the levels of Cr and BUN in the model group and allopurinol group were increased significantly, while WESJ significantly reduced their levels in the serum of HUA mice. The results were consistent with the H&E staining results of renal cortex tissue, that is, high concentration of UA caused kidney injury, which was intensified by allopurinol, and WESJ improved renal excretory dysfunction in model mice.

### 3.5. Effects of WESJ on Kidney Histopathology in Hyperuricemic Mice 

**Figure 5:**
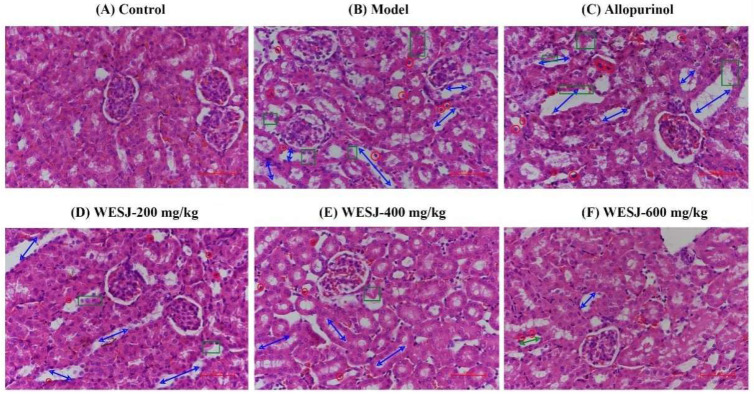
Renal sections for H&E staining (magnification, 400×). Note: renal tubule dilation (blue arrows); pyknotic or necrotic nuclei (red circles); lesion, necrosis, or exfoliation of the tubular epithelium (green squares).

Figure 5 shows H&E staining results of renal cortex tissue in different groups of mice. The morphology of the renal cortex of control group mice was normal, with no histopathological change. Mice in the model group exhibited severe renal structural impairments, which manifested as obvious expansion of renal tubules, pyknosis of the nucleus, and nuclear necrosis in severe cases, as well as damage to the renal tubular epithelium. The allopurinol group displayed further renal injury, suggesting that allopurinol is severely nephrotoxic. After administration of WESJ for treatment, it was found that with the increase of the administration dose, the kidney damage in mice was gradually alleviated, for which improvement was manifested in the relief of renal tubular dilatation and the reduction of renal tubular epithelial cell damage. These results suggest that WESJ ameliorated renal damage in HUA mice in a dose-dependent manner and exerted a nephroprotective effect.

### 3.6. Determination of Chemical and Serum Components of WESJ

**Figure 6:**
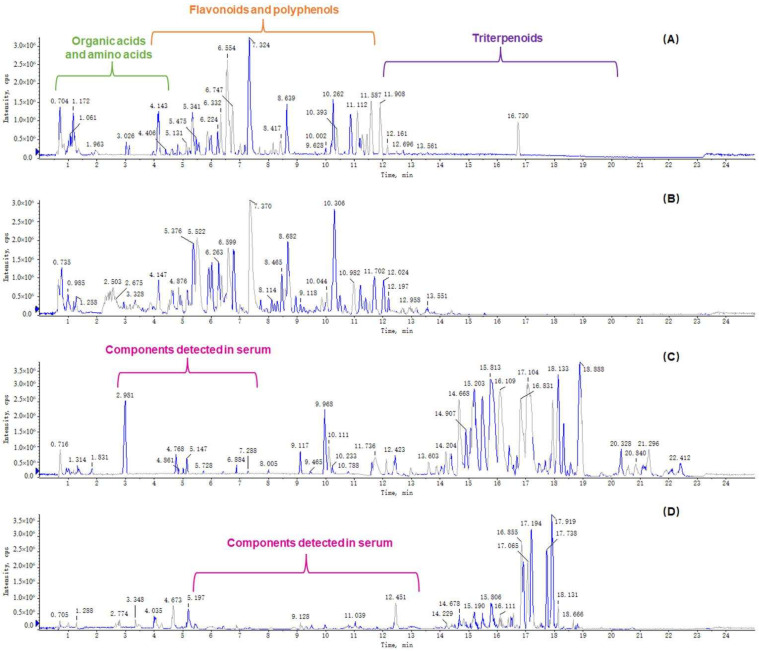
Base peak chromatograms. (**A**) WESJ in positive ion mode; (**B**) WESJ in negative ion mode; (**C**) serum sample collected at 2.0 h after dosing in positive ion mode; (**D**) serum sample collected at 2.0 h after dosing in negative ion mode.

**Figure 7:**
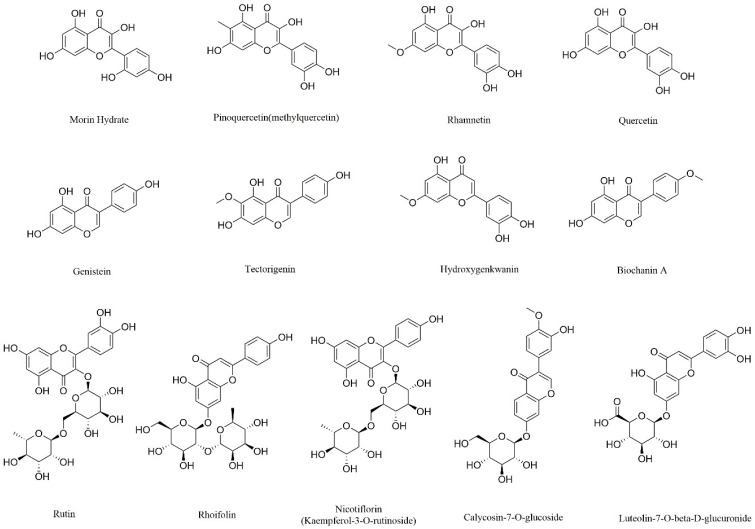
Chemical structural of representative flavonoids and polyphenols bioactive components in WESJ.

A total of 214 compounds were identified in WESJ, including 152 flavonoids and polyphenols, 33 organic acids, 13 triterpenoids, 5 alkaloids, and 11 other compounds. Blood components of WESJ were mainly investigated based on the prototype. A total of 23 compounds were identified, including 16 flavonoids and polyphenols, five triterpenoids, and two other compounds. The consequences of the identified compounds are listed in Table S1. Base peak chromatograms of WESJ in positive and negative ion mode, and serum sample collected at 2.0 h after dosing in positive and negative ion mode, are shown in Figure 6. In terms of the peak time, the peak time of organic acids was mainly in the first 3 min, the peak time of flavonoids and polyphenols was concentrated about 3–12 min, while the peak time of triterpenoids was about 12–19 min. The chemical structure of representative flavonoids and polyphenols bioactive components in WESJ is shown in Figure 7. The results indicated that flavonoids or polyphenols may be the main bioactive components of *Sophora japonica* and could be developed as dietary supplements for the treatment of HUA [16].

## 4. Conclusions

This study showed that WESJ can effectively reduce serum UA levels and ameliorate the damage to the kidneys in HUA mice at a dose of 200–600 mg/kg. The underlying therapeutic mechanism is to inhibit XOD activity. UPLC-Q-TOF-MS/MS method was used to determine the chemical components in WESJ and blood-entering prototype components, which were flavonoids and polyphenols. WESJ has the potential to be used in the treatment of HUA, in which flavonoids and polyphenols, as the main bioactive components, can be developed as dietary supplements. This study will provide a meaningful reference for the research and development of plant-based foods for the treatment of HUA. However, there are still some limitations in this study. The study on the mechanism of anti-HUA only investigated the influence of UA production process, that is, the inhibition of XOD activity, but not the influence on UA transporters. In addition, the activity of the identified compounds still needs further study.

## Data Availability

Data are contained within the article and supplemental materials.

## Supplementary Materials

The following supporting information can be downloaded at: https://www.mdpi.com/article/10.3390/foods11233772/s1, Table S1: Serum components identified 2 h after WESJ administration.
