# Supplementary material for: Bioactivity and Component Analysis of Water Extract of Sophora japonica against Hyperuricemia by Inhibiting Xanthine Oxidase Activity
Source: Foods. 2022 Nov 23;11(23):3772. doi: 10.3390/foods11233772 (PMC9740820; doi:10.3390/foods11233772)
Supplement: Supplementary file 1 [file foods-11-03772-s001.zip › foods-2003078-supplementary-revised (1).pdf]

Supplementary Table S1. Serum components identified 2 h after WESJ administration.

No.	Component Name	Area	Retention Time (min)	Adduct / Charge	Formula	Precursor Mass	Found At Mass	Mass Error (ppm)
1	2-[3-[3,4-dihydroxy-4-(hydroxymethyl)oxolan-2-yl]oxy-4,5-dihydroxy-6-(hydroxymethyl)oxan-2-yl]oxy-3-methylpyran-4-one or 3-[6-[[3,4-dihydroxy-4-(hydroxymethyl)oxolan-2-yl]oxymethyl]-3,4,5-trihydroxyoxan-2-yl]oxy-2-methylpyran-4-one	9.89×10^{4}	2.72	$[M+H]^+$	$C_{17}H_{24}O_{12}$	421.134	421.1345	1.1
2	Licoagroside B	1.11×10^{6}	4.13	$[M+H]^+$	$C_{18}H_{24}O_{12}$	433.134	433.1344	0.8
3	Quercetin-glc	6.49×10^{4}	5.35	$[M+H]^+$	$C_{21}H_{20}O_{12}$	465.103	465.1032	0.9
4	Rutin	1.71×10^{6}	5.36	$[M-H]^-$	$C_{27}H_{30}O_{16}$	609.146	609.1456	−0.9
5	Morin hydrate	1.90×10^{5}	5.55	$[M+H]^+$	$C_{15}H_{10}O_{7}$	303.050	303.0504	1.7
6	Kaempferol-3-O-rutinoside	5.08×10^{4}	5.62	$[M-H]^-$	$C_{27}H_{30}O_{15}$	593.151	593.1513	0.2
7	Kaempferol-O-glc-rha	1.79×10^{5}	5.89	$[M-H]^-$	$C_{27}H_{30}O_{15}$	593.151	593.151	−0.4
8	Rhoifolin	5.31×10^{4}	5.99	$[M-H]^-$	$C_{27}H_{30}O_{14}$	577.156	577.1554	−1.4
9	methylquercetin	1.34×10^{5}	6.00	$[M+H]^+$	$C_{16}H_{12}O_{7}$	317.066	317.0656	0.2

10	Isorhamentin-3-O-glc-rha	5.66×10^{5}	6.01	$[M-H]^{-}$	$C_{28}H_{32}O_{16}$	623.162	623.1611	−1.0
11	Luteolin-7-O-β-D-glucuronide	9.29×10^{5}	6.09	$[M+H]^{+}$	$C_{21}H_{18}O_{12}$	463.087	463.0874	0.7
12	Rhamnetin	9.45×10^{4}	6.56	$[M-H]^{-}$	$C_{16}H_{12}O_{7}$	315.051	315.0507	−1.0
13	Quercetin	1.46×10^{5}	7.35	$[M+H]^{+}$	$C_{15}H_{10}O_{7}$	303.050	303.050	0.4
14	Calycosin-7-O-glc	4.43×10^{4}	7.70	$[M+H]^{+}$	$C_{22}H_{22}O_{10}$	447.129	447.128	−1.2
15	Genistein	1.20×10^{6}	8.10	$[M-H]^{-}$	$C_{15}H_{10}O_{5}$	269.046	269.0453	−0.9
16	Tectorigenin	1.18×10^{5}	8.18	$[M-H]^{-}$	$C_{16}H_{12}O_{6}$	299.056	299.0556	−1.6
17	Hydroxygenkwanin	1.11×10^{5}	8.28	$[M-H]^{-}$	$C_{16}H_{12}O_{6}$	299.056	299.0558	−1.1
18	Madecassoside	2.07×10^{4}	8.38	$[M-H]^{-}$	$C_{48}H_{78}O_{20}$	973.501	973.5013	0
19	Ginsenoside-Ro	1.48×10^{5}	10.42	$[M-H]^{-}$	$C_{48}H_{76}O_{19}$	955.491	955.4908	0
20	Biochanin A	1.72×10^{4}	10.63	$[M-H]^{-}$	$C_{16}H_{12}O_{5}$	283.061	283.0604	−2.7
21	Dehydrosoyasaponin I	1.22×10^{5}	11.36	$[M-H]^{-}$	$C_{48}H_{76}O_{18}$	939.496	939.4955	−0.4
22	Kaikasaponin III	1.17×10^{5}	13.18	$[M-H]^{-}$	$C_{48}H_{78}O_{17}$	925.517	925.5172	0.7
23	Kaikasaponin I	1.14×10^{5}	13.56	$[M-H]^{-}$	$C_{42}H_{68}O_{13}$	779.459	779.4585	−0.3
